# The application of EPSiT in pilonidal sinus disease: an international Delphi consensus study endorsed by the Association of Laparoscopic Surgeons of Great Britain and Ireland (ALSGBI)

**DOI:** 10.1007/s10151-025-03191-7

**Published:** 2025-07-30

**Authors:** H. K. Sekhon Inderjit Singh, P. Meinero, F. C. Campanile, A. Quddus, R. Rajaganeshan, J. Warusavitarne, V. Dotlacil, A. Bhargava, P. Giordano, A. Pini Prato, V. Shatkar, P. Jalali, V. C. Halahakoon, G. Gallo, M. Milone, S. Mantoo, C. A. Leo, C. Esposito, M. Farghaly, T. Arulampalam, N. Pawa

**Affiliations:** 1 Department of Surgery, Barking, Havering and Redbridge NHS Trust, Romford, Greater London, UK; 2 Department of Surgery and Cancer, Imperial College London, London, UK; 3 Department of Surgery, Montallegro Clinic, Genoa, Italy; 4 Division of General Surgery, Ospedale San Giovanni Decollato, ASL Viterbo, Andosilla, Civita Castellana, Italy; 5 Department of Surgery, Homerton Healthcare NHS Trust, Hackney, London, UK; 6 Department of Surgery, Mersey and West Lancashire NHS Trust, Prescot, UK; 7 Department of Surgery, St Mark’s Hospital, London, UK; 8 Department of Paediatric Surgery, Second Faculty of Medicine, Charles University and Motol University Hospital, Prague, Czech Republic; 9 Department of Colorectal Surgery, Whipps Cross Hospital, London, UK; 10 Department of Surgery, The Royal London Hospital, Barts NHS Trust, London, UK; 11 Umberto Bosio Center for Digestive Diseases, The Children Hospital, AOU SS Antonio e Biagio e Cesare Arrigo, Alessandria, Italy; 12 Research Institute for Gastroenterology and Liver Diseases, Shahid Beheshti University of Medical Sciences, Tehran, Iran; 13 Department of Surgery, East Suffolk and North Essex Foundation Trust, Colchester, UK; 14 Department of Surgery, Sapienza University of Rome, Rome, Italy; 15 Department of Clinical Medicine and Surgery, University of Naples ‘Frederico II’, Naples, Italy; 16 Department of Surgery, Khoo Teck Puat Hospital, Singapore, Singapore; 17 Division of Paediatric Surgery, Frederico II University of Naples, Naples, Italy; 18 General Surgery Department, Farwaniya Hospital, Al Farwaniyah, Kuwait; 19 Colorectal Unit, Ain Shams University, Cairo, Egypt; 20 School of Medicine, Anglia Ruskin University, Chelmsford, UK; 21 Department of Surgery, Chelsea and Westminster NHS Foundation Trust, West Middlesex University Hospital, Twickenham Road, Isleworth, London, TW7 6AF UK

**Keywords:** Pilonidal disease, Chronic, Endoscopic, Primary, Recurrent, Adult, Paediatric, Consensus

## Abstract

**Background:**

Endoscopic pilonidal sinus treatment (EPSiT) is a novel, minimally invasive surgical technique that has shown promise in the treatment of pilonidal sinus disease. Despite the apparent benefits and call for increased use, widespread uptake has been slow. This study aims to gather and understand expert international opinions on EPSiT and develop recommendations for its application in the surgical community.

**Methods:**

Expert international panellists were identified and recruited to participate. A three-round modified Delphi consensus consisting of 43 questions regarding the application of EPSiT was posed. A combination of a five-point Likert scale, binary ‘yes/no’ scale and multiple-choice questions was used. The consensus threshold was set at 70% agreement. When consensus was not achieved or further insight was required, statement questions were posed. The study has been performed in accordance with ACcurate COnsensus Reporting Document (ACCORD) explanation and elaboration guidelines.

**Results:**

Twenty experts from six countries participated in all rounds, with a 100% response rate. Our experts agreed on 28 statements including: the absence of absolute contraindications to EPSiT; administering intravenous induction antibiotics routinely but not post-operative oral antibiotics; recommending laser epilation; offering re-EPSiT to the informed patient after first and second procedure failures; and that EPSiT should be incorporated into surgical training programmes.

**Conclusions:**

This is the first study to provide an international expert consensus on the specific application of EPSiT in primary and recurrent adult and paediatric patients with pilonidal sinus disease. The findings of this study contribute to the development of protocols for EPSiT in pilonidal sinus disease management, addressing key areas of consensus and controversy and promoting procedure uptake.

**Supplementary Information:**

The online version contains supplementary material available at 10.1007/s10151-025-03191-7.

## Introduction

Chronic pilonidal sinus disease (PD) has long been a challenging condition for both surgeons and patients, with traditional surgical techniques such as open excision and off-midline primary closure (Bascoms’, Karydakis, Limberg flap, etc.), often resulting in issues such as poor wound healing, wound infection and breakdown, long periods of post-operative pain, disease recurrence and poor quality of life [1–4]. However, two promising, similar, minimally invasive alternatives were introduced: endoscopic pilonidal sinus treatment (EPSiT) in 2013 [5] and video-assisted ablation of pilonidal sinus (VAAPS) in 2014 [6].

Both consist of a diagnostic phase followed by a therapeutic phase. With the patient in the prone position and a single dose of antibiotic prophylaxis administered, a 5-mm incision is made around the spontaneously discharging sinus. A fistuloscope (hysteroscope in VAAPS) is then introduced through this opening, and the sinus cavity and fistula tracts are identified. All hairs inside the sinus are removed with forceps; the fistula cavity and tracts are then cauterised with an electrode, and epithelial and granulation tissue is then removed with an endobrush or Volkmann spoon. The procedure is performed entirely under direct vision. Glycine/mannitol 1% solution is infused throughout the EPSiT procedure, while saline solution is used in VAAPS [5, 6]. The post-operative management of the small wound involves a light dressing with no packing and once-daily wound washing using a syringe with saline solution for at least 2 weeks [5]. Compared with conventional surgery, these endoscopic techniques have a significantly reduced rate of complications (wound infection, wound dehiscence, seroma, haematoma), post-procedure pain and hospital stay and a faster return to daily activities. However, the risk of recurrence has not been shown to be significantly different [4–10].

Despite its potential benefits, its widespread uptake has been slow, particularly in the United Kingdom (UK), where it is not routinely offered in every hospital. This is likely multifactorial as a result of evolving evidence, lack of guidelines, concerns regarding its success in both primary and recurrent disease, consultant surgeon training or experience and failure to incorporate it into the surgical training programme for registrars. In addition, where uptake has occurred, modifications from the original technique have been proposed and preferred. These include the use of oral post-operative antibiotics [11], post-operative wound management with negative pressure dressings [12], phenol application into the tract [13] or the use of laser instead of cauterisation [14]. This can lead to ambiguity and further hesitation in uptake. Data from the UK PITSTOP cohort, designed to reflect real-world clinical settings, highlighted that EPSiT was employed in only 7% of cases from 2019 to 2022 [15]. Furthermore, a consensus statement published by the Italian Society of Colorectal Surgery on the management of PD agreed that minimally invasive endoscopic procedures should be the treatment of choice in cases of limited disease and that the treatment of recurrent disease should not differ from that of primary disease [16]. Similar practice priorities resulted from the PITSTOP study [17]. Taken together, it is predicted that EPSiT uptake should increase.

We are embarking on a Delphi consensus aimed at understanding the nuances of the surgical technique employed by experts in this procedure and developing recommendations and a protocol to better standardise and inform the uptake of EPSiT in treating PD in adults and children. On this basis, the Association of Laparoscopic Surgeons of Great Britain and Ireland (ALSGBI) was approached. The association agreed that this research would benefit their members, and the surgical community in general, resulting in endorsement. A Delphi design was chosen because it has been shown to be favourable in healthcare where there is ambiguity and limited knowledge [18, 19]. In this manuscript, EPSiT and VAAPS are considered interchangeable, referring to the technique described in the second paragraph, and unless specified, they will refer to both adult and paediatric groups.

## Methodology

### Study facilitators

H.K.S.I.S., T.A., and N.P. are the study facilitators directing this exercise with endorsement by the ALSGBI. T.A. and N.P. have been practising EPSiT for more than 5 years under the National Health Service and private practice in the UK. On the basis of expertise, experience and gaps in the literature, both recognised the need for the development of a standardised protocol to improve the uptake of EPSiT. H.K.S.I.S. is a senior trainee surgeon in the UK with an interest in EPSiT and conducting research.

### Expert selection

Twenty-nine surgeons practising EPSiT were contacted by study facilitators and invited to participate via email. Fifteen surgeons in the UK were identified through a Storz database of recurrent EPSiT kit users. Thirteen internationally based surgeons who had published research on the use of this technique were also approached. One expert panellist recommended that another panellist be approached for inclusion. The aim was to recruit at least 20 expert panellists to ensure a sufficient representation, understanding that EPSiT is not practised by the majority of general/colorectal surgeons. Fifteen or more participants was previously shown to be sufficient to provide reliable data [20]. Baseline information regarding the number of EPSiT procedures, years of practice, frequency and outcome data was obtained to ensure expert status. An expert is defined as a surgeon who has performed > 50 EPSiT cases.

### Delphi process

**Fig. 1:**
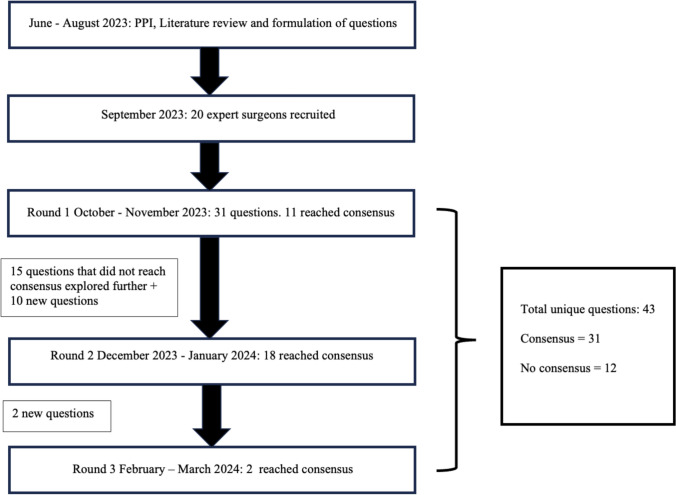
Flowchart reviewing the modified Delphi protocol. PPI: Patient participation

The modified Delphi process began in June 2023 and was completed in March 2024 (Fig. 1). Five patients with PD were consulted regarding the aims and methodology of this consensus. All patient participants provided positive feedback regarding the need for this study. No negative feedback or recommendations were made. A systematic review of the literature was initially performed using MEDLINE and EMBASE databases from January 2012 to June 2023. Twenty key papers highlighting the most up-to-date evidence regarding EPSiT in adults and children were identified by the study facilitators and distributed via email to all panellists to ensure standardisation of knowledge prior to the distribution of the online questionnaires (Supplement 2).

### Questionnaires

The three study facilitators designed the questionnaires, which consisted of statements divided into five domains: ‘Pre-operatively’, ‘Intra-operatively’, ‘Post-operatively’, ‘Modified EPSiT/Other’ and ‘Training’. The questionnaire was distributed via email, with each email containing a personalised link to the online questionnaire on TypeForm. A combination of a five-point Likert scale, binary ‘yes/no’ scale and multiple-choice questions was used. In each round, the experts were allowed to suggest further statements that they felt should be explored. Definitions for specific terms were specified in each round and are provided in Supplementary material.

The consensus threshold was set at 70% agreement for all rounds. Where consensus was not achieved, panellists were encouraged to submit free-text comments for further insight. All responses remained anonymous to all expert panellists, including T.A. and N.P. as study facilitators. Only H.K.S.I.S., who did not participate in the questionnaires, was not blinded to any of the responses. At the end of each round, H.K.S.I.S. collated and anonymised the results, which were then sent to the panellists, summarising statements that had and had not achieved consensus. Further rounds contained new questions posed by the panellists and further explored the questions that had not achieved consensus in the prior round. Bi-weekly reminder emails were sent to the panellists to encourage responses. A total of 43 unique statements were explored over three rounds.

A total of three rounds were conducted as no new questions were proposed by the panellists at the end of the third round, the majority of questions had reached consensus and the study facilitators were concerned about participation fatigue. Three rounds is typical for a Delphi census study. Piloting of the online questionnaire was not necessary as, in this digital age, healthcare professionals are familiar with using online platforms for research. In addition, none of the panellists raised concerns regarding understanding of the questionnaire when prompted. This consensus is reported in accordance with ACCORD guidelines [21] (Supplementary material).

### Statistical analysis

Simple quantitative analysis of data was performed.

## Results

### Our panel

Twenty consultant general surgeons from six countries (the UK, Italy, Iran, the Czech Republic, Kuwait and Singapore) agreed to participate. Their EPSiT practice patterns, self-reported EPSiT outcomes and experience with modified EPSiT techniques are reported in Table 1.

**Table 1:** Summary of expert panel practice and outcomes. *N* is number of panellists unless otherwise specified

Category	Subcategory	*N* (%)
Patient population	Adults only	12 (60%)
	Adults and children	7 (35%)
	Children only	1 (5%)
Sector of practice	Public and private	13 (65%)
	Public only	5 (25%)
	Private only	2 (10%)
Years performing EPSiT	> 5 years	13 (65%)
	1–5 years	7 (35%)
Total procedures performed	> 200	6 (30%)
	> 100	5 (25%)
	> 50	9 (45%)
Monthly case volume	> 3	14 (70%)
	1–3	4 (20%)
	< 1	2 (10%)
Self-reported success rate	Primary simple disease	Mean: 83.2% (range: 70%–96%)
	Complex recurrent disease	Mean: 70.7% (range: 40%–100%)
Experience with laser techniques	No experience with sinus laser therapy	18 (90%)
	No experience with laser-assisted EPSiT	14 (70%)

### Survey rounds

A 100% response rate was achieved for all questions in all rounds. The statements and consensus rates are summarised in Tables 2 and 3. There were no deviations from our planned protocol.

**Table 2:** Achieved consensus

Category	No.	Statement	Consensus rate
Pre-operatively	1	The ideal method of treating primary or recurrent, simple or complex pilonidal disease would be a technique that achieves short hospital stays, low complication rates, low recurrence rates, good cosmesis, quick return to daily life/work and is cheap	Agreement—95%
	2	Does EPSiT achieve this?	Agreement—95%
	3	Are there absolute contraindications to the use of EPSiT?	Disagreement—85%
	4	Is Complex disease a contraindication to the use of EPSiT?	Disagreement—95%
	5	Is Recurrent disease without a previous flap a contraindication to the use of EPSiT?	Disagreement—100%
	6	Is Recurrent disease with a previous flap a contraindication to the use of EPSiT?	Disagreement—85%
	7	Is Male gender a contraindication to the use of EPSiT?	Disagreement—100%
	8	Is Extremes of age a contraindication to the use of EPSiT?	Disagreement—85%
	9	Are ‘Patient Characteristics’ a contraindication to the use of EPSiT?	Disagreement—85%
	10	Do you agree that an individual with a short < 1 cm tract or an open wound where the tract will not distend appropriately is a relative contra-indication to EPSiT?	Agreement—75%
	11	Besides the above factors (4–10) are there any other relative or absolute contra-indications?	Disagreement—100%
	12	Is there a role for MRI pre-operatively in excluding other pathology? Yes/No	Agreement—70%
	13	Accepting that short-term recurrence rates are similar, should EPSiT be the preferred technique to treat primary and recurrent pilonidal sinus disease in adults compared to traditional techniques (Bascoms, Karydakis, Excision and Closure)?	Agreement—90%
	14	Accepting that short-term recurrence rates are similar, should EPSiT be the preferred technique in children to treat primary and recurrent pilonidal sinus disease compared to traditional techniques (Bascoms, Karydakis, Excision and Closure)?	Agreement—80%
Intra-operatively	15	In your practice, is stat intravenous induction antibiotic routinely given to prevent wound infection and/or recurrence in EPSIT?	Agreement—80%
	16	In your practice, are the buttocks routinely separated with tape? Or will this run the risk of compressing and missing small secondary tracts?	Agreement—80%
Post-operatively	17	Is there a role for MRI post-operatively in supporting conservative management if the wound fails to heal or disease recurs? In this time healing may occur. Yes/No	Disagreement—75%
	18	In your practice, do you routinely prescribe a course of oral antibiotics (5–7 days) post EPSiT to decrease wound infection, promote wound healing and decrease recurrence?	Disagreement—70%
	19	What oral antibiotics do you prescribe?	Agreement—83.3%
	20	Would you recommend prolonged irrigation for delayed wound healing or persistent discharge	Agreement—100%
	21	Should additional adjuncts to promote wound healing be consistently used?	Agreement—75%
	22	In your practice, do you routinely advise laser epilation (hair removal) pre-procedure and/or post-procedure?	Agreement—85%
	23	Why does EPSiT not work on some patients i.e. failure to heal or recurrence – disease related?	Agreement—80%
	24	Why does EPSiT not work on some patients i.e. failure to heal or recurrence – technique related?	Agreement—75%
	25	How do you manage failure to heal/recurrence after EPSiT on primary disease (1 failed EPSiT procedure)?	Agreement—80%
	26	How do you manage failure to heal/recurrence after EPSiT on recurrent disease (2 failed procedures, one of which is EPSiT)?	Agreement—70%
	27	How do you manage failure to heal/recurrence after EPSiT on recurrent disease (2 failed EPSiT procedures)?	Agreement—75%
Modified EPSiT/Other	28	Are there scenarios where Cautery-Phenol EPSiT is preferred to standard (P)EPSiT?	Unsure/No opinion—80%
	29	Costs: EPSiT is cost-effective long-term compared to traditional surgical techniques	Agreement—95%
Training	30	Do you feel that EPSiT should be incorporated routinely into surgical registrar (or equivalent) training?	Agreement—100%
	31	Do you routinely train surgical registrars (or equivalent) under your tutelage how to perform EPSiT?	Agreement—100%

**Table 3:** Did not achieve consensus

Category	No.	Statement	Consensus rate
Pre-operatively	1	Are there relative contraindications to the use of EPSiT?	Agreement—50%
	2	Is there a role for MRI pre-operatively in mapping pilonidal disease where the disease is complex or recurrent? Yes/No	Agreement—50%
Intra-operatively	3	Is the endobrush strictly only used after ablation as described in the original technique?	Agreement—50%
Post- operatively	4	What is the ideal duration of post-procedure wound irrigation with saline?	10–15 days—60%
	5	If you agree that additional adjuncts should be used to promote wound healing which do you recommend: Select as many as you wish A—Manuka Honey B—NPWT C—PRP D—Antibiotic infused dressings E—Oxygen enriched dressings	NPWT—35%
	6	Would you recommend any other form of hair removal routinely pre or post-op in both adolescent and adult populations including A—Waxing B—Shaving C—Over the counter creams D—All of the above	All of the above—40%
	7	When would you recommend hair removal post-operatively in both adolescent and adult populations?	Immediately post-operatively—40%
	8	What is your definition of ‘recurrence’ and ‘Failure to heal’?	Agreement—50%
	9	Why does EPSiT not work on some patients i.e. failure to heal or recurrence – patient related?	Agreement—65%
Modified EPSiT/Other	10	Given the limited current evidence, are there scenarios where Laser assisted EPSiT (LEPSiT) is preferred to standard EPSiT?	Agreement—65%
	11	Should patients with complex pilonidal disease be managed/referred to joint plastic-general surgical clinics to optimise their management?	Agreement—50%
	12	Regarding the above, is this a service you currently offer in your practice?	Agreement—40%

### Section 1: Pre-operatively

Nineteen of the 20 experts agreed that the ideal treatment for simple or recurrent PD is one that is minimally invasive, has low complication rates, is cheap, and is associated with good cosmesis and quick return to daily life, and that EPSiT can achieve this (agreement: 95%). Experts agreed that there is no absolute contraindication for EPSiT (85%) and that patient characteristics, complex disease and recurrent disease with or without a flap were not contraindications (range: 85–100%). Patient characteristics include age, gender, obesity, sedentary lifestyle, smoking history, family history of PD and work that involves constant irritation of the natal cleft. Experts also agreed (75%) that patients with a short < 1 cm tract or an open wound that would not allow the tract to distend properly is a relative contraindication to the procedure. There was 100% agreement that no other factors should be considered before performing EPSiT.

Consensus was not achieved regarding the use of preoperative magnetic resonance imaging (MRI) to map the disease where the disease is complex or recurrent (50%). The panel agreed that MRI was only useful in excluding other pathologies such as fistula-in-ano (70%). It is not useful in supporting conservative management within which time healing may occur (75%).

The majority of experts agreed that EPSiT be the preferred technique to treat primary and recurrent PD in adults (90%) and children (80%) compared with traditional techniques, given that recurrence rates are similar.

### Section 2: Intra-operatively

Experts agreed that single-dose intravenous induction antibiotics should routinely be administered to prevent wound infection and/or recurrence in EPSIT (80%) and that the patients’ buttocks should be routinely separated with tape (80%). Consensus was not achieved regarding the importance of performing brushing after ablation (50%).

### Section 3: Post-operatively

Expert panellists agreed that routine prescription of a course of oral antibiotics (5–7 days) post-EPSiT is not required to decrease wound infection, promote wound healing and decrease the risk of recurrence (70%). If oral antibiotics are required, Gram-positive cover such as co-amoxiclav is agreed on (83.3%). The majority (60%) would recommend to patients that they irrigate their wound with saline flushes for 10–15 days; however, this did not reach consensus. Complete agreement was reached regarding indications to extend saline irrigation, that is, the persistence of wound discharge/delayed healing.

Seventy-five percent agreed that additional adjuncts to promote wound healing should be consistently used; however, the adjunct could not be agreed on with 7/20 recommending negative pressure wound therapy, 3/20 recommending manuka honey, and a few others recommending adjuncts such as platelet-rich plasma (PRP) (1/20), antibiotic-infused dressing (1/20), oxygen-enriched dressings (2/20) and weekly bedside curettage with silver nitrate for ooze (1/20).

With regards to hair removal, agreement was reached regarding the recommendation of laser epilation pre-procedure and/or post-procedure (85%). However, consensus was not reached regarding recommending other forms of hair removal including waxing alone (0/20), shaving alone (5/20), over-the-counter creams alone (2/20) or use of all of the above (8/20). Consensus was also not reached regarding the timing to commence hair removal. The majority of experts suggest commencing hair removal immediately post-operatively (8/20; 40%).

The definitions of ‘failure to heal’ and ‘recurrence’ were not agreed upon, with four experts suggesting persistence or recurrence of symptoms respectively at 3 months (20%), ten experts at 3–6 months (50%) and six at 6 months (30%). Consensus regarding the reason why some patients fail to heal or recur was reached for ‘Disease-related’ (80%) and ‘Technique-related’ factors (75%), where disease-related factors are defined as complex disease with multiple tracts/sinuses, caudal disease and recurrent disease and technique-related factors are defined as failure to achieve complete hair control or sufficient brushing/diathermy of the tract. Consensus regarding the reason why some patients fail to heal or recur for patient-related factors such as high body mass index (BMI), current or ex smoking status and hirsute was not reached (65%).

Regarding further management in patients with failure to heal or recurrence post-EPSiT, experts agreed that, for both primary and recurrent disease, they would offer a further EPSiT procedure (> 70% agreement). At the time of recurrence after the second EPSiT, 75% of experts would offer a third EPSiT procedure.

### Section 4: Modified EPSiT/other

Consensus was not reached for the preferred use of laser-assisted endoscopic pilonidal sinus treatment (LEPSIT) over standard EPSiT, with the majority suggesting that there are no scenarios where they would prefer it (65%). On further probing using free text, this is primarily because of a lack of evidence as well as no laser facility within departments (ten panellists). Two surgeons who had attempted laser felt that it was more expensive, with the same to worse outcomes for their patient cohort. Panellists were ‘Unsure’ or had ‘No opinion’ regarding the preferred use of cautery-phenol EPSiT over standard EPSiT (80%).

Experts agree that EPSiT is a cost-effective treatment in the long term compared with traditional surgical techniques for PD (95%).

Fifty percent of experts think that patients with complex PD should be managed in joint plastic–general surgical clinics to optimise their management, with 40% of their institutions offering this service.

**Fig. 2:**
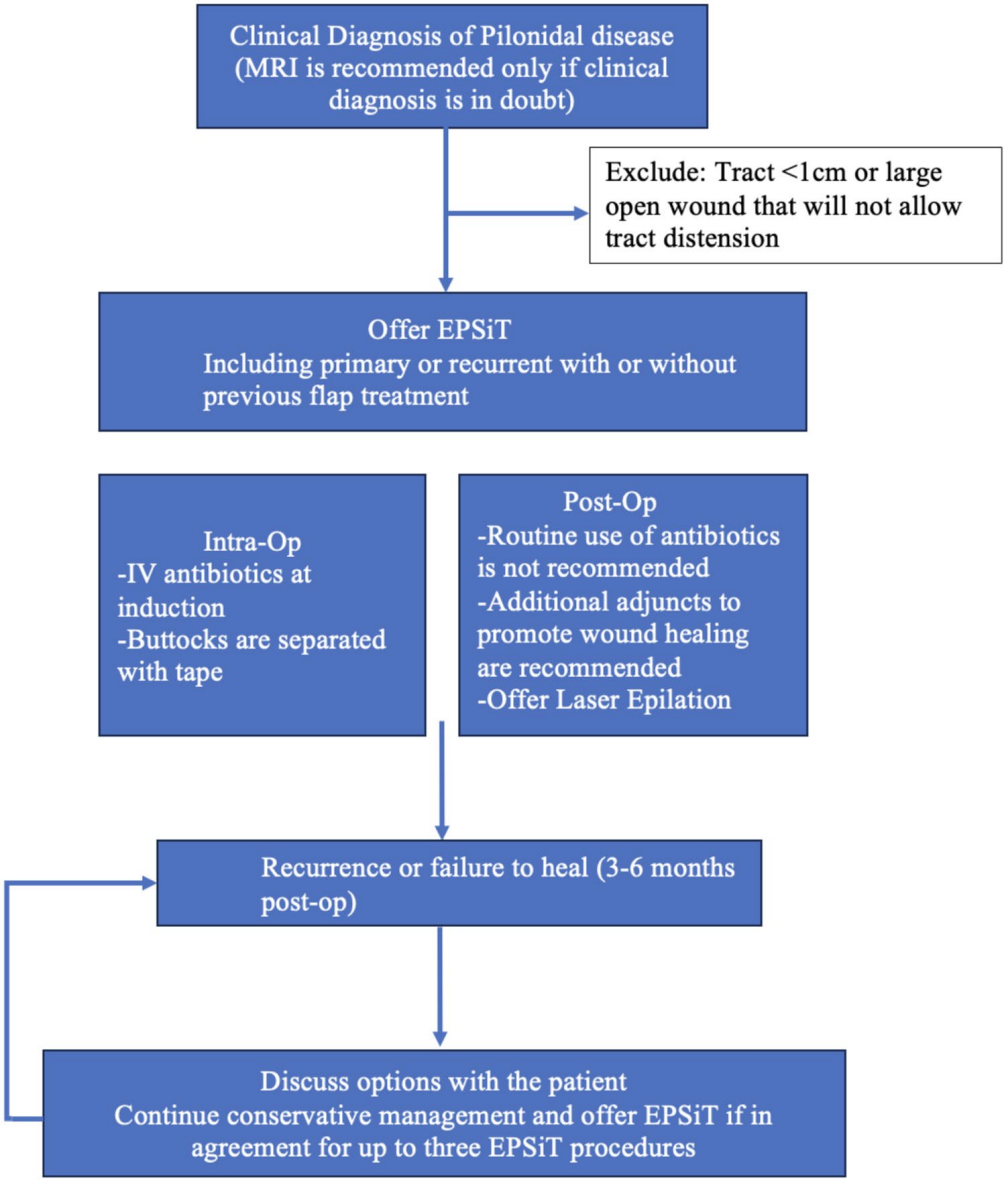
Consensus on the application of EPSiT in pilonidal disease

On the basis of these results, experts agreed on an algorithm for the application of EPSiT in PD, which was developed and is highlighted in Fig. 2.

### Section 5: Training

Unanimous agreement was reached regarding the necessary incorporation of EPSiT into surgical registrar (or equivalent) training, with all experts routinely training those under their tutelage. Free-text discussion regarding the proposed criteria to measure proficiency in EPSiT ranged from observational clinical human reliability analysis-style training to ensuring that a minimum number of level 4 procedure-based assessments are completed. The suggested learning curve ranged from 10 or fewer procedures (5/20) to 20–30 procedures (11/20), and less than 50 procedures (4/20).

## Discussion

This is the first Delphi consensus aimed at developing a protocol for employing EPSIT in adult and paediatric patients with chronic PD (Fig. 2). We are satisfied that our panel are experts in this procedure, given that they have performed at least 50 procedures. The learning curve for EPSiT has been suggested to be approximately 15 procedures [22]. In addition, their recurrence rates in simple primary and complex disease reflect that in the literature [3–8]. There is no need to differentiate between adult and paediatric populations as the risk factors are the same, the surgical technique is the same and results reported in the literature are similar between the populations [23].

Every surgeon is aware that patient selection is as important as operative technique with regard to the resulting outcomes. The consensus panel’s assertion that patient and disease factors should not contraindicate EPSiT is an important finding that aligns with emerging evidence advocating its broad applicability [24]. Observational studies have provided contradictory evidence regarding the increased risk of recurrence or failure of EPSiT secondary to these factors apart from tract laterality from the midline [24, 25]. These could be dealt with by using the additional openings as a point of access rather than relying only on the midline sinus and is likely why our experts did not consider this aspect of tract morphology a contraindication. We emphasise instead specifically avoiding only cases with tracts < 1 cm (i.e. short tracts) or wide external openings as retention of the infiltrating solution and distension of the tract are challenging in these instances and more likely to result in failure. These cases should make up a very small proportion of patients with PD.

In the spectrum of PD, we recommend that EPSiT be the preferred technique to treat both primary and recurrent disease, irrespective of previous treatment, given that recurrence rates are similar and that EPSiT has low complication rates compared with conventional treatment [3–8, 23]. This is supported by a recent discrete choice experiment suggesting that patients are willing to trade the risk of infection/recurrence for a faster recovery, especially those over the age of 30 years [26]. Pitfalls of this choice experiment include the option of ranking only five treatment options, of which EPSiT was not one, and outcomes beyond infection/recurrence and recovery, such as pain or cosmesis, were not assessed. In a purely paediatric cohort, significant improvement in objective measures of QoL including return to sport, school and social life after EPSiT compared with open surgery has been shown. The authors also demonstrate subjective maintenance of a good to very good QOL after EPSiT; however, there was no comparison group [27]. Furthermore, Hinksman et al. have also highlighted the viability and safety of offering repeated EPSiT after treatment failure with no increased technical difficulty or complications [24]. It is also because of a shift to this repeated minimally invasive approach that the majority of our experts do not believe that patients with complex PD would routinely benefit from management in joint plastic–general surgical clinics.

Although MRI offers high soft tissue resolution capable of mapping PD, our panel’s recommendation to limit its use to cases of diagnostic uncertainty only is prudent. Its use in supporting conservative management, should the wound fail to heal or the disease recur, has not been studied before and is understandably not recommended by our experts. The same vain is taken with our recommendation to continue to separate the buttocks using tape: Only anecdotal accounts have proposed that this may compress smaller tracts, resulting in their being missed with EPSiT.

Other notable areas of controversy pertain to the sequence of brushing and ablation during EPSiT, two key steps, as well as the role of antibiotics. Although brushing after ablation theoretically enhances the fibrotic response [5, 6], no direct comparisons have been made. When trialled by a team in Turkey, brushing before and after ablation did not seem to confer a difference in average operative time, wound healing rates and recurrence [14, 28]. Antibiotics can be administered at induction, post-operatively regionally or locally to the wound in order to decrease wound complications, improve wound healing and decrease recurrence. Despite the absence of evidence specific to EPSiT, the consensus panel recommends the administration of intravenous antibiotics during induction. This reflects the broader surgical practice of using prophylactic antibiotics to reduce the risk of infection. However, the panel does not endorse routine postoperative antibiotic use, citing inconsistent evidence from studies on conventional pilonidal surgery [29–36]. For cases requiring postoperative antibiotics, regimens with Gram-positive coverage such as co-amoxiclav, for 5–7 days, are advised. These recommendations align with broader trends in antimicrobial stewardship, aiming to minimize unnecessary antibiotic use while addressing infection risk in high-risk cases. Future studies should focus on clarifying the role of targeted perioperative antibiotics in reducing wound complications and recurrence rates in EPSiT.

Within the spectrum of PD, there has been considerable variation in use of aids with anti-bacterial, anti-inflammatory and/or growth factor stimulating properties to promote wound healing. These include manuka honey [37, 38], PRP [39–41] and negative-pressure wound therapy [42–45]. In addition, oxygen-infused dressings have been shown to significantly improve the closure of chronic wounds, such as diabetic foot ulcers [46]. Specific to EPSiT, Esposito et al. account for the use of laser epilation with oxygen-enriched oil based dressings as a significant contributing factor to their improved healing times, low wound complication rates and low recurrence rates [47].They strongly advocate for this to become the standardised protocol for wound care following EPSiT in children [47]. A comparison of oxygen-enriched dressings with antibiotic and hyaluronic acid dressings in EPSiT suggests that the former also significantly reduces the need for post-procedure analgesia [22]. Giordano and colleagues advocate for the use of NPWT post-EPSiT as it was felt to be particularly useful as a barrier against faecal contamination and was more practical for the patient [12]. Complete healing was achieved in only 84.6% of their 13 patient cohort. No data on the use of PRP in EPSiT have been published, but there is concern that its use may increase the risk of abscess formation, as the plasma may be less able to drain effectively from minimally invasive wounds [38], in addition to being more costly and time-consuming owing to the need for repeated applications. Incorporating these aids post-EPSiT naturally prevents or makes the need to irrigate the wound with saline, as originally described by Meinero, redundant. This largely explains why a consensus was not reached regarding recommending this to patients. However, if saline irrigation was recommended, the presence of ongoing wound discharge or delayed healing is an indicator to extend the recommended duration of irrigation beyond 10–15 days. The presence of body hair at the natal cleft is a risk factor for recurrence and wound complications in PD [48]. Therefore, controlling the hair pre- and post-operatively should decrease recurrence rates. Traditional methods of hair control involve shaving, waxing or chemical depilation; however, they can be difficult to perform within the deep natal cleft or have poor long-term compliance [49–51], especially considering that patients ideally need to control hair growth for 2 years [48, 49]. In addition, some studies suggest shaving specifically may increase recurrence rates [52], likely owing to its propensity to promote ingrown hairs. Newer laser epilation technology has the advantage of overcoming all these issues [50]. Importantly, cessation of hair growth can be maintained for half a year post treatment [51, 53–55]. Esposito et al. showed that not only was laser epilation safe and well tolerated by paediatric patients undergoing EPSIT both pre- and post-procedure, it also significantly decreased wound complication and recurrence rates [47]. Similar positive findings have been reported in adult populations undergoing traditional surgical treatments [49, 50]. Where possible, trusts should offer patients laser epilation as an adjunct to EPSiT to improve outcomes.

Only two small cohort studies comparing LEPSIT with EPSIT (*n* < 100), to our knowledge, have been published. Outcomes suggest no difference in return to daily activities, wound complication rates and recurrence rates between the two procedures [14, 56]. EPSIT in addition was significantly less expensive than LEPSIT [14]. A further single cohort study (*n* = 42) comparing cautery-phenol EPSiT with LEPSiT showed no significant difference in the time to return to daily activities, time to wound healing and recurrence rates [57]. The lack of number and high-quality evidence reflects why consensus was either not reached or why the majority of experts were unsure or had no opinion regarding the preferred use of LEPSiT or cautery-phenol EPSiT over EPSiT, respectively. Interestingly, two of our experts had attempted laser treatment, with similar outcomes to Gulcu and Ozturk, and hence did not find it of greater benefit than EPSiT [14].

The PITSTOP study highlighted the disappointing ongoing use of major excisional surgery in PD in the UK [15]. This is despite guideline recommendations [16]. Barriers to the incorporation of EPSiT into training include the availability of expertise, standardisation of technique and documented competency. Enhancing consultant uptake, supported by a census like this one, should aid surgical societies and other stakeholders to advocate for its incorporation into training programmes. Although biased to a degree, it is predictable that our experts unanimously concur that EPSiT be included into surgical registrar (or equivalent) training and already do this themselves (100%). Regarding competency, approximately half (11/20, 55%) of the experts felt that trainees could achieve competency at around 20–30 procedures, slightly higher than that proposed in the literature [22]. With the broad applicability of EPSiT, these numbers should not be hard to achieve.

There are limitations to our study. We identified local experts through a Storz database of recurrent EPSiT kit users, which can be criticised as unconventional and lead to selection bias, but it was felt to be appropriate by the study facilitators as Storz is the only local supplier of this kit. However, our methodology involved effectively anonymising responses, aiming to minimise response bias. Of note, our methodology is also easily reproducible, with a 100% response rate from panellists. When it came to understanding factors determining recurrence or failure to heal, these were grouped into patient-, technique- and disease-related factors. This was intentional, as the Delphi process focussed on determining consensus around key domains rather than an exhaustive variable-by-variable analysis. We acknowledge that this is an area where future work could provide more granular insights. Although aiming to be generalisable by including experts from different countries, some countries may not have access to the equipment required, leading to limited global applicability. No cost analysis studies have been performed comparing EPSiT with other conventional procedures, although costs over the long term are predicted to be less owing to reduced postoperative care requirements, shorter hospital stays and quicker return to work or school in this young, active population [3]. This is a census in an area where further studies are still needed to clarify areas of ambiguity such as long-term recurrence rates (> 5 years) for EPSiT and other minimally invasive procedures or consensus on the definitions of ‘recurrence’ or ‘failure to heal’ on which we measure success. However, with the current evidence base that exists, our protocol will be a good starting point for surgeons wishing to offer this treatment to patients and stakeholders incorporate it into training programmes.

## Conclusions

Our Delphi consensus study provides valuable insights into the current practices and perceptions surrounding EPSiT in the management of PD. By elucidating key management considerations, areas of consensus and controversy, and providing a standardised algorithm, our findings aim to inform clinical practice guidelines and facilitate the widespread adoption of EPSiT, ultimately improving patient outcomes and quality of life.

## Supplementary Information

Below is the link to the electronic supplementary material.

Supplementary file1 (DOCX 48 KB)

## Data Availability

Data are provided within the manuscript or supplementary information files.

## References

[1] Abdulraheem,A. Boutros M. Pilonidal disease in 2022: Where do we stand? Seminars in Colon and Rectal Surgery. 2022; 10.1016/j.scrs.2022.100910

[2] Enriquez-Navascues JM, Emparanza JI, Alkorta M, Placer C (2014) Meta-analysis of randomized controlled trials comparing different techniques with primary closure for chronic pilonidal sinus. Tech Coloproctol 18:863–72. 10.1007/s10151-014-1149-5 24845110 10.1007/s10151-014-1149-5

[3] Lee WG, Short C, Zhong A, Vojvodic V, Sundin A, Spurrier RG, Wang KS, Pelayo JC (2024) Outcomes of pediatric pilonidal disease treatment: excision with off-midline flap reconstruction versus endoscopic pilonidal sinus treatment. Pediatr Surg Int 40(1):46. 10.1007/s00383-023-05629-1 38294551 10.1007/s00383-023-05629-1 PMC10830615

[4] Milone M, Velotti N, Manigrasso M, Vertaldi S, Di Lauro K, De Simone G, Cirillo V, Maione F, Gennarelli N, Sosa Fernandez LM, De Palma GD (2020) Long-term results of a randomized clinical trial comparing endoscopic versus conventional treatment of pilonidal sinus. Int J Surg (London, England) 74:81–85. 10.1016/j.ijsu.2019.12.033 10.1016/j.ijsu.2019.12.033 31926328

[5] Meinero P, Mori L, Gasloli G (2014) Endoscopic pilonidal sinus treatment (E.P.Si.T). Tech Coloproctol 18:389–392. 10.1007/s10151-013-1016-9 23681300 10.1007/s10151-013-1016-9

[6] Milone M, Musella M, Di Spiezio Sardo A, Bifulco G, Salvatore G, Sosa Fernandez LM, Bianco P, Zizolfi B, Nappi C, Milone F (2014) Video-assisted ablation of pilonidal sinus: a new minimally invasive treatment–a pilot study. Surgery 155(3):562–566. 10.1016/j.surg.2013.08.021 24300343 10.1016/j.surg.2013.08.021

[7] Chen S, Dai G, Liu P, Zhao X, Zhang J, Yang C, Xu X, Wang L, Chen W, Wang M, Zhang D (2022) Comparative analysis on the effect of the endoscopic versus conventional treatment for pilonidal sinus: a meta-analysis of controlled clinical trials. Medicine 101(45):e31767. 10.1097/MD.0000000000031767 36397424 10.1097/MD.0000000000031767 PMC9666099

[8] Milone M, Fernandez LM, Musella M, Milone F (2016) Safety and efficacy of minimally invasive video-assisted ablation of pilonidal sinus: a randomized clinical trial. JAMA Surg 151(6):547–553. 10.1001/jamasurg.2015.5233 26819186 10.1001/jamasurg.2015.5233

[9] Tien T, Athem R, Arulampalam T (2018) Outcomes of endoscopic pilonidal sinus treatment (EPSiT): a systematic review. Tech Coloproctol 22(5):325–331. 10.1007/s10151-018-1803-4 29850944 10.1007/s10151-018-1803-4

[10] Foti N, Passannanti D, Libia A, Campanile FC (2021) A minimally invasive approach to pilonidal disease with endoscopic pilonidal sinus treatment (EPSiT): a single-center case series with long-term results. Tech Coloproctol 25(9):1045–1054. 10.1007/s10151-021-02477-w 34110535 10.1007/s10151-021-02477-w

[11] Azhough R, Azari Y, Taher S, Jalali P (2021) Endoscopic pilonidal sinus treatment: a minimally invasive surgical technique. Asian J Endoscopic Surg 14(3):458–463. 10.1111/ases.12893 10.1111/ases.12893 33185031

[12] Giordano P, Schembari E, Keshishian K, Leo CA (2021) Negative pressure-assisted endoscopic pilonidal sinus treatment. Tech Coloproctol 25(6):739–743. 10.1007/s10151-021-02431-w 33755853 10.1007/s10151-021-02431-w

[13] Gecim IE, Goktug UU, Celasin H (2017) Endoscopic pilonidal sinus treatment combined with crystalized phenol application may prevent recurrence. Dis Colon Rectum 60(4):405–407. 10.1097/DCR.0000000000000778 28267008 10.1097/DCR.0000000000000778

[14] Gulcu B, Ozturk E (2022) Endoscopic pilonidal sinus treatment vs. laser-assisted endoscopic pilonidal sinus treatment: short-term results from a retrospective case-matched study. Tech Coloproctol 26(4):271–277. 10.1007/s10151-021-02568-8 35025023 10.1007/s10151-021-02568-8

[15] Brown SR, Hind D, Strong E, Bradburn M, Din F, Lee E, Lund J, Moffatt C, Morton J, Senapati A, Jones H, Lee MJ, PITSTOP Management Group (2024) Real-world practice and outcomes in pilonidal surgery: pilonidal sinus treatment studying the options (PITSTOP) cohort. Br J Surg 111(3):009. 10.1093/bjs/znae009 10.1093/bjs/znae009 PMC10941257 38488204

[16] Milone M, Basso L, Manigrasso M, Pietroletti R, Bondurri A, La Torre M, Milito G, Pozzo M, Segre D, Perinotti R, Gallo G (2021) Consensus statement of the Italian society of colorectal surgery (SICCR): management and treatment of pilonidal disease. Tech Coloproctol 25(12):1269–1280. 10.1007/s10151-021-02487-8 34176001 10.1007/s10151-021-02487-8 PMC8580911

[17] Lee MJ, Lee E, Bradburn M, Hind D, Strong EB, Din F, Wysocki AP, Lund J, Moffatt C, Morton J, Senapati A, Jones H, Brown SR, PITSTOP Management Group and the PITSTOP Validators (2024) Research and practice priorities in pilonidal sinus disease: a consensus from the PITSTOP study. Colorectal Disease offi j Assoc Coloproctol Great Britain Ireland. 10.1111/codi.16946.Advanceonlinepublication.10.1111/codi.16946 10.1111/codi.16946 PMC11683161 38671581

[18] Cresswell KM, Panesar SS, Salvilla SA, Carson-Stevens A, Larizgoitia I, Donaldson LJ, Bates D, Sheikh A (2013) World Health Organization’s (WHO) safer primary care expert working group global research priorities to better understand the burden of iatrogenic harm in primary care: an international Delphi exercise. PLoS Med 10(11):e1001554. 10.1371/journal.pmed.1001554 24260028 10.1371/journal.pmed.1001554 PMC3833831

[19] McKenna HP (1994) The Delphi technique: a worthwhile research approach for nursing? J Adv Nurs 19(6):1221–1225. 10.1111/j.1365-2648.1994.tb01207.x 7930104 10.1111/j.1365-2648.1994.tb01207.x

[20] Lord A, Brown G, Abulafi M, Bateman A, Frankel W, Goldin R, Gopal P, Kirsch R, Loughrey MB, Märkl B, Moran B, Puppa G, Rasheed S, Shimada Y, Snaebjornsson P, Svrcek M, Washington K, West N, Wong N, Nagtegaal I (2021) Histopathological diagnosis of tumour deposits in colorectal cancer: a Delphi consensus study. Histopathology 79(2):168–175. 10.1111/his.14344 33511676 10.1111/his.14344

[21] Logullo P, van Zuuren EJ, Winchester CC, Tovey D, Gattrell WT, Price A, Harrison N, Goldman K, Chisholm A, Walters K, Blazey P (2024) Accurate Consensus Reporting Document (ACCORD) explanation and elaboration: Guidance and examples to support reporting consensus methods. PLoSmedicine 21(5):e1004390. 10.1371/journal.pmed.1004390 10.1371/journal.pmed.1004390 PMC11198995 38709851

[22] Parente G, Ruspi F, Thomas E, Di Mitri M, Cravano SM, D’Antonio S, Gargano T, Lima M (2023) Endoscopic pilonidal sinus treatment: preliminary results, learning curve and comparison with standard open approach. Children 10(6):1063. 10.3390/children10061063 37371294 10.3390/children10061063 PMC10297686

[23] Esposito C, Montaruli E, Autorino G, Mendoza-Sagaon M, Escolino M (2021) Pediatric endoscopic pilonidal sinus treatment (PEPSiT): What we learned after a 3-year experience in the pediatric population. Updates Surg 73:2331–2339 34021885 10.1007/s13304-021-01094-4 PMC8606398

[24] Hinksman M, Naidu S, Loon K, Grundy J (2022) Long-term efficacy of endoscopic pilonidal sinus treatment: a single-centre Australian experience. ANZ J Surg 92(5):1142–1148. 10.1111/ans.17666 35347830 10.1111/ans.17666

[25] Maione F, D’Amore A, Milone M, Vertaldi S, Anoldo P, Chini A, Sorrentino C, Marello A, Cantore G, Maione R, D’Angelo S, D’Alesio N, De Simone G, Servillo G, De Palma GD, Manigrasso M (2023) Endoscopic approach to complex or recurrent pilonidal sinus: a retrospective analysis. Int Wound J 20(4):1212–1218. 10.1111/iwj.13980 36271666 10.1111/iwj.13980 PMC10031245

[26] Wickramasekera N, Strong E, Shackley P, Callaghan T, Lee M, Hind D, Brown S, PITSTOP Project Management Group and PITSTOP Collaborators (2023) Patient preferences for pilonidal sinus treatments: a discrete choice experiment survey. Colorectal Dis Off J Assoc Coloproctol Great Britain Ireland. 10.1111/codi.16482.Advanceonlinepublication.10.1111/codi.16482 10.1111/codi.16482 36636796

[27] Esposito C, Lepore B, Cerulo M, Borgogni R, Del Conte F, Coppola V, Di Mento C, Carulli R, Cardone R, Cortese G, Esposito G, Escolino M (2023) Quality of life of pediatric patients operated for pilonidal sinus disease. Eur J Pediatr 182(1):25–30. 10.1007/s00431-022-04678-3 36348071 10.1007/s00431-022-04678-3 PMC9829630

[28] Gulcu B, Ozturk E (2021) Endoscopic pilonidal sinus treatment: rapid recovery, satisfactory success, and high quality of life. Surg Laparoscopy Endoscopy Percutaneous Tech 31(6):711–715. 10.1097/SLE.0000000000000974 10.1097/SLE.0000000000000974 34310558

[29] Sondenaa K, Nesvik I, Gullaksen FP et al (1995) The role of cefoxitin prophylaxis in chronic pilonidal sinus treated with excision and primary suture. J Am Coll Surg 180:157–160 7850048

[30] Kundes MF, Cetin K, Kement M et al (2016) Does prophylactic antibiotic reduce surgical site infections after rhomboid excision and Limberg flap for pilonidal disease: a prospective randomized double blind study. Int J Colorectal Dis 31:1089–1091 26525054 10.1007/s00384-015-2425-1

[31] Chaudhuri A, Bekdash BA, Taylor AL (2006) Single-dose metronidazole vs. 5-day multi-drug antibiotic regimen in excision of pilonidal sinuses with primary closure: a prospective, randomized, double-blinded pilot study. Int J Colorectal Dis 21:688–692 16362397 10.1007/s00384-005-0064-7

[32] Kronborg O, Christensen K, Zimmermann-Nielsen C (1985) Chronic pilonidal disease: a randomized trial with a complete 3-year follow-up. Br J Surg 72:303–304 3886069 10.1002/bjs.1800720418

[33] Mavros MN, Mitsikostas PK, Alexiou VG, Peppas G, Falagas ME (2013) Antimicrobials as an adjunct to pilonidal disease surgery: a systematic review of the literature. Eur J Clin Microbiol Infect Dis 32:851–858 23380885 10.1007/s10096-013-1830-z

[34] Yetim I, Ozkan OV, Dervişoglu A, Erzurumlu K, Canbolant E (2010) Effect of gentamicin-absorbed collagen in wound healing in pilonidal sinus surgery: a prospective randomized study. J Int Med Res 38(3):1029–1033 20819439 10.1177/147323001003800329

[35] Rao MM, Zawislak W, Kennedy R, Gilliland R (2010) A prospective randomised study comparing two treatment modalities for chronic pilonidal sinus with a 5-year follow-up. Int J Colorectal Dis 25:395–400 19823853 10.1007/s00384-009-0804-1

[36] Andersson RE, Lukas G, Skullman S, Hugander A (2010) Local administration of antibiotics by gentamicin-collagen sponge does not improve wound healing or reduce recurrence rate after pilonidal excision with primary suture: a prospective randomized controlled trial. World J Surg 34:3042–3048 20734046 10.1007/s00268-010-0763-2

[37] Abet E, Jean MH, Greilsamer T, Planche L, Maurice F, Brau-Weber AG, Denimal F (2023) The value of honey dressings in pilonidal cyst healing: a prospective randomized single-center trial. Tech Coloproctol 27(9):721–727. 10.1007/s10151-022-02740-8 36598614 10.1007/s10151-022-02740-8

[38] Salehi V, Yavari Barhaghtalab MJ, Mehrabi S, Iraji A, Sadat SA, Yusefi SH, Malekzadeh JM (2022) Does application of honey improve surgical outcome in pilonidal cyst excision with secondary intention healing? A prospective randomized placebo-controlled clinical trial. Perioperative medicine 11(1):1. 10.1186/s13741-021-00237-w 35000582 10.1186/s13741-021-00237-w PMC8744332

[39] Brewer CF, Correia IFS, Miranda BH (2022) Platelet-rich plasma (PRP) for sacrococcygeal pilonidal disease: an updated systematic review and meta-analysis. World J Surg 46(12):2910–2918. 10.1007/s00268-022-06711-w 36064868 10.1007/s00268-022-06711-w

[40] Khan QI, Baig H, Al Failakawi A, Majeed S, Khan M, Lucocq J (2022) The effect of platelet-rich plasma on healing time in patients following pilonidal sinus surgery: a systematic review. Cureus 14(8):e27777. 10.7759/cureus.27777 36106230 10.7759/cureus.27777 PMC9450803

[41] Boztug CY, Karaagac Akyol T, Benlice C, Koc MA, Doganay Erdogan B, Ozcebe OI, Kuzu MA, Akyol C (2021) Platelet-rich plasma treatment improves postoperative recovery in patients with pilonidal sinus disease: a randomized controlled clinical trial. BMC Surg 21(1):373. 10.1186/s12893-021-01370-5 34670534 10.1186/s12893-021-01370-5 PMC8529773

[42] Danne J, Gwini S, McKenzie D, Danne P (2017) A retrospective study of pilonidal sinus healing by secondary intention using negative pressure wound therapy versus alginate or gauze dressings. Ostomy Wound Manage 63(3):47–53 28355137

[43] Carnali M, Ronchi R, Finocchi L, Meletani T, Capesciotti SS, Paggi B (2016) Retrospective study on the use of negative pressure wound therapy in the treatment of pilonidal cysts (sinus pilonidalis) operated on using an open technique or complicated by dehiscence of the surgery site through sepsis. Acta Vulnol 14:24–39

[44] Nakamichi M, Ogino A, Onishi K (2020) Less invasive treatment for the pilonidal sinus combined use of negative-pressure wound therapy. Eur J Plast Surg 43:75–78. 10.1007/s00238-019-01560-8

[45] Biter LU, Beck GM, Mannaerts GH, Stok MM, van der Ham AC, Grotenhuis BA (2014) The use of negative-pressure wound therapy in pilonidal sinus disease: a randomized controlled trial comparing negative-pressure wound therapy versus standard open wound care after surgical excision. Dis Colon Rectum 57:1406–1411. 10.1097/DCR.0000000000000240 25380007 10.1097/DCR.0000000000000240

[46] Fitzpatrick E, Holland OJ, Vanderlelie JJ (2018) Ozone therapy for the treatment of chronic wounds: a systematic review. Int Wound J 15(4):633–644 29536625 10.1111/iwj.12907 PMC7949634

[47] Esposito C, Del Conte F, Esposito G, Coppola V, Cerulo M, Escolino M (2020) Standardization of pre- and postoperative management using laser epilation and oxygen-enriched oil-based gel dressing in pediatric patients undergoing pediatric endoscopic pilonidal sinus treatment (PEPSiT). Lasers Surg Med. 10.1002/lsm.23318 32964496 10.1002/lsm.23318

[48] Karydakis GE (1991) Easy and successful treatment of pilonidal sinus after explanation of its causative process. Aust N Z J Surg 62(5):385–389. 10.1111/j.1445-2197.1992.tb07208.x 10.1111/j.1445-2197.1992.tb07208.x 1575660

[49] Lopez JJ, Cooper JN, Fischer BA, Gonzalez DO, Deans KJ, Minneci PC (2017) Safety and tolerability of laser hair depilation in pilonidal disease: a pilot study. Surg Infect 18(8):890–893. 10.1089/sur.2017.153 10.1089/sur.2017.153 29016243

[50] Pronk AA, Eppink L, Smakman N, Furnee EJB (2018) The effect of hair removal after surgery for sacrococcygeal pilonidal sinus disease: a systematic review of the literature. Tech Coloproctol 22(1):7–14. 10.1007/s10151-017-1722-9 29185064 10.1007/s10151-017-1722-9

[51] Odili J, Gault D (2002) Laser depilation of the natal cleft an aid to healing the pilonidal sinus. Ann R Coll Surg Engl 84(1):29–32 11890622 PMC2503766

[52] Petersen S, Wietelmann K, Evers T, Hüser N, Matevossian E, Doll D (2009) Long-term effects of postoperative razor epilation in pilonidal sinus disease. Dis Colon Rectum 52:131–134 19273968 10.1007/DCR.0b013e3181972505

[53] Landa N, Aller O, Landa-Gundin N, Torrontegui J, Azpiazu JL (2005) Successful treatment of recurrent pilonidal sinus with laser epilation. Dermato Surg Off Public Am Soc Dermatol Surg 31(6):726–728. 10.1111/j.1524-4725.2005.31601 10.1111/j.1524-4725.2005.31601 15996432

[54] Benedetto AV, Lewis AT (2005) Pilonidal sinus disease treated by depilation using an 800 nm diode laser and review of the literature. Dermato Surg Off Public Am Soc Dermatol Surg 31(5):587–591. 10.1111/j.1524-4725.2005.31169 10.1111/j.1524-4725.2005.31169 15962749

[55] Badawy EA, Kanawati MN (2009) Effect of hair removal by Nd:YAG laser on the recurrence of pilonidal sinus. J Eur Acad Dermatol Venereol 23(8):883–886. 10.1111/j.1468-3083.2009.03147.x 19586514 10.1111/j.1468-3083.2009.03147.x

[56] Ersavas C, Erginel B, Yanar F, Azamat İF, Taskesen F, Soysal FG (2023) Endoscopic pilonidal sinus treatment (EPSIT) versus sinus laser therapy (SiLaT) for sacrococcygeal pilonidal sinus. Wideochirurgia i inne techniki maloinwazyjne Videosurg Other Miniinvasive Tech. 18(1):144–148 37064557 10.5114/wiitm.2022.124206 PMC10091928

[57] Dönmez M, Uludag M (2022) Evaluation of the Early Outcomes of Laser-Endoscopic Pilonidal Sinus Treatment Combination and Comparison With the Combination of Cautery-Phenol-Endoscopic Pilonidal Sinus Treatment. Cureus 14(7):e26948. 10.7759/cureus.26948 10.7759/cureus.26948 PMC9378937 35989794

