# Supplementary material for: The application of EPSiT in pilonidal sinus disease: an international Delphi consensus study endorsed by the Association of Laparoscopic Surgeons of Great Britain and Ireland (ALSGBI)
Source: Tech Coloproctol. 2025 Jul 30;29(1):155. doi: 10.1007/s10151-025-03191-7 (PMC12310914; doi:10.1007/s10151-025-03191-7)
Supplement: Supplementary file 1 — Supplementary file1 (DOCX 48 KB) [file 10151_2025_3191_MOESM1_ESM.docx]

**Search strategy**

The Medline and Embase databases (January 2012 to June 2023) were searched using the following search strategy:

1. Pilonidal
2. Pilonidal sinus
3. Endoscopic Pilonidal sinus treatment
4. EPSiT
5. PEPSiT
6. Adjuncts
7. Laser
8. Antibiotics
9. 1 OR 2
10. 3 or 4
11. 9 AND 10
12. 5 OR 11
13. 6 OR 7 OR 8 AND 12

1     Pilonidal Sinus/ or Pilonidal.mp. (2733)
2     PEPSiT.mp. (15)
3     EPSiT.mp. (34)
4     Endoscopic pilonidal sinus treatment.mp. (48)
5     adjuncts.mp. (7528)
6     laser.mp. or Lasers/ (369727)
7     antibiotics.mp. or Anti-Bacterial Agents/ (584841)
8     1 or 2 or 3 or 4 (2820)
10     8 and 5 (53)
11     8 and 6 (61)
12     8 and 7 (12)
13     10 or 11 or 12 (126)
14     limit 13 to (english language and yr="2012 - 2023") (101)

Studies selected as landmark or key studies for distribution to panellists

(n = 20)

**Identification**

**Included**

Record Titles screened

(n = 69)

Abstracts sought for retrieval

(n = 54)

Reports assessed for eligibility by study facilitators

(n = 48)

Records identified: 101

**Screening**

**Identification of studies via Medline and Embase**

Duplicates

(n = 32)

Reports excluded, irrelevant to EPSiT: 15

Reports excluded, irrelevant to EPSiT: 6

Reports excluded, as not considered landmark or key studies on adjuncts, hair removal or modified EPSiT: 28

*PRISMA Flow Diagram*

*From:*  Page MJ, McKenzie JE, Bossuyt PM, Boutron I, Hoffmann TC, Mulrow CD, et al. The PRISMA 2020 statement: an updated guideline for reporting systematic reviews. BMJ 2021;372:n71. doi: 10.1136/bmj.n71

For more information, visit: <http://www.prisma-statement.org/>

References of the 20 studies sent to the expert panellists:

Included in the reference list:

1. Lee, W. G., Short, C., Zhong, A., Vojvodic, V., Sundin, A., Spurrier, R. G., Wang, K. S., & Pelayo, J. C. (2024). Outcomes of pediatric pilonidal disease treatment: excision with off-midline flap reconstruction versus endoscopic pilonidal sinus treatment. *Pediatric surgery international*, *40*(1), 46. <https://doi.org/10.1007/s00383-023-05629-1>
2. Milone, M., Velotti, N., Manigrasso, M., Vertaldi, S., Di Lauro, K., De Simone, G., Cirillo, V., Maione, F., Gennarelli, N., Sosa Fernandez, L. M., & De Palma, G. D. (2020). Long-term results of a randomized clinical trial comparing endoscopic versus conventional treatment of pilonidal sinus. *International journal of surgery (London, England)*, *74*, 81–85.
3. Meinero, P., Mori, L. & Gasloli, G (2014). Endoscopic pilonidal sinus treatment (E.P.Si.T.). *Tech Coloproctol* 18, 389–392. <https://doi.org/10.1007/s10151-013-1016-9>
4. Milone, M., Musella, M., Di Spiezio Sardo, A., Bifulco, G., Salvatore, G., Sosa Fernandez, L. M., Bianco, P., Zizolfi, B., Nappi, C., & Milone, F. (2014). Video-assisted ablation of pilonidal sinus: a new minimally invasive treatment--a pilot study. *Surgery*, *155*(3), 562–566. <https://doi.org/10.1016/j.surg.2013.08.021>
5. Chen, S., Dai, G., Liu, P., Zhao, X., Zhang, J., Yang, C., Xu, X., Wang, L., Chen, W., Wang, M., & Zhang, D. (2022). Comparative analysis on the eﬀect of the endoscopic versus conventional treatment for pilonidal sinus: A meta-analysis of controlled clinical trials. Medicine, 101(45), e31767. <https://doi.org/10.1097/MD.0000000000031767>
6. Milone, M., Fernandez, L. M., Musella, M., & Milone, F. (2016). Safety and Efficacy of Minimally Invasive Video-Assisted Ablation of Pilonidal Sinus: A Randomized Clinical Trial. *JAMA surgery*, *151*(6), 547–553. <https://doi.org/10.1001/jamasurg.2015.5233>
7. Foti, N., Passannanti, D., Libia, A., & Campanile, F. C. (2021). A minimally invasive approach to pilonidal disease with endoscopic pilonidal sinus treatment (EPSiT): a single-center case series with long-term results. *Techniques in coloproctology*, *25*(9), 1045–1054. <https://doi.org/10.1007/s10151-021-02477-w>
8. Giordano, P., Schembari, E., Keshishian, K., & Leo, C. A. (2021). Negative pressure-assisted endoscopic pilonidal sinus treatment. Techniques in coloproctology, 25(6), 739–743. h;ps://doi.org/10.1007/s10151-021-02431-w
9. Gecim, Ibrahim Ethem M.D.1; Goktug, Utku Ufuk M.D.1; Celasin, Haydar M.D.2. Endoscopic Pilonidal Sinus Treatment Combined With Crystalized Phenol Application May Prevent Recurrence. Diseases of the Colon & Rectum 60(4):p 405-407, April 2017. | DOI: 10.1097/DCR.0000000000000778
10. Gulcu, B., & Ozturk, E. (2022). Endoscopic pilonidal sinus treatment vs. laser-assisted endoscopic pilonidal sinus treatment: short-term results from a retrospective case-matched study. Techniques in coloproctology, 26(4), 271–277. hnps://doi.org/10.1007/s10151-021-02568-8
11. Milone, M., Basso, L., Manigrasso, M., Pietroletti, R., Bondurri, A., La Torre, M., Milito, G., Pozzo, M., Segre, D., Perinotti, R., & Gallo, G. (2021). Consensus statement of the Italian society of colorectal surgery (SICCR): management and treatment of pilonidal disease. *Techniques in coloproctology*, *25*(12), 1269–1280. <https://doi.org/10.1007/s10151-021-02487-8>
12. Parente, G., Ruspi, F., Thomas, E., Di Mitri, M., Cravano, S. M., D'Antonio, S., Gargano, T., & Lima, M. (2023). Endoscopic Pilonidal Sinus Treatment: Preliminary Results, Learning Curve and Comparison with Standard Open Approach. *Children (Basel, Switzerland)*, *10*(6), 1063. <https://doi.org/10.3390/children10061063>

Esposito, C.; Montaruli, E.; Autorino, G.; Mendoza-Sagaon, M.; Escolino, M. (2021) Pediatric endoscopic pilonidal sinus treatment (PEPSiT): What we learned after a 3-year experience in the pediatric population. *Updates Surg.* 73, 2331–2339

Maione, F., D'Amore, A., Milone, M., Vertaldi, S., Anoldo, P., Chini, A., Sorrentino, C., Marello, A., Cantore, G., Maione, R., D'Angelo, S., D'Alesio, N., De Simone, G., Servillo, G., De Palma, G. D., & Manigrasso, M. (2023). Endoscopic approach to complex or recurrent pilonidal sinus: A retrospective analysis. *International wound journal*, *20*(4), 1212–1218. <https://doi.org/10.1111/iwj.13980>

1. Esposito, C., Lepore, B., Cerulo, M., Borgogni, R., Del Conte, F., Coppola, V., Di Mento, C., Carulli, R., Cardone, R., Cortese, G., Esposito, G., & Escolino, M. (2023). Quality of life of pediatric patients operated for pilonidal sinus disease. *European journal of pediatrics*, *182*(1), 25–30. <https://doi.org/10.1007/s00431-022-04678-3>
2. Esposito, C., Del Conte, F., Esposito, G., Coppola, V., Cerulo, M., & Escolino, M. (2020). Standardization of Pre- and Postoperative Management Using Laser Epilation and Oxygen-Enriched Oil-Based Gel Dressing in Pediatric Patients Undergoing Pediatric Endoscopic Pilonidal Sinus Treatment (PEPSiT). *Lasers in surgery and medicine*, 10.1002/lsm.23318. Advance online publication. <https://doi.org/10.1002/lsm.23318>
3. Ersavas, C., Erginel, B., Yanar, F., Azamat, İ. F., Taskesen, F., & Soysal, F. G. (2023). Endoscopic pilonidal sinus treatment (EPSIT) versus sinus laser therapy (SiLaT) for sacrococcygeal pilonidal sinus. *Wideochirurgia i inne techniki maloinwazyjne = Videosurgery and other miniinvasive techniques*, *18*(1), 144–148.
4. Dönmez, M., & Uludag, M. (2022). Evaluation of the Early Outcomes of Laser-Endoscopic Pilonidal Sinus Treatment Combination and Comparison With the Combination of Cautery-Phenol-Endoscopic Pilonidal Sinus Treatment. *Cureus*, *14*(7), e26948. <https://doi.org/10.7759/cureus.26948>

Not included in the reference list:

Romaniszyn, M., Swirta, J. S., & Walega, P. J. (2020). Long-term results of endoscopic pilonidal sinus treatment vs Limberg flap for treatment of difficult cases of complicated pilonidal disease: a prospective, nonrandomized study. *Colorectal disease : the official journal of the Association of Coloproctology of Great Britain and Ireland*, *22*(3), 319–324. <https://doi.org/10.1111/codi.14857>

Manigrasso, M., Velotti, N., Sosa Fernandez, L. M., Vertaldi, S., Maione, F., Gennarelli, N., Dinuzzi, V. P., Musella, M., De Palma, G. D., & Milone, M. (2021). Endoscopic Approach to Recurrent Pilonidal Sinus: A Retrospective Analysis. *Journal of laparoendoscopic & advanced surgical techniques. Part A*, *31*(1), 1–5. <https://doi.org/10.1089/lap.2020.0252>

Definitions:

Complex PD- A patient with recurrent PD or primary PD with multiple tracts, branching tracts or caudal disease

Disease related factors impacting failure or recurrence – Refers to complex disease with multiple tracts/sinuses, caudal disease, and recurrent disease

Expert – Is an expert is defined as a surgeon who has performed >50 EPSiT cases.

Failure to heal – Is the persistence of symptoms at a defined time point post operatively

Open wound- Refers to a previously unhealed or partially healed wound with no tunnelled tract

Patient Characteristics impacting failure or recurrence - Refers to age, gender, obesity, sedentary lifestyle, smoking history, family history of PD and work that involves constant irritation of the natal cleft

Primary PD- A patient with PD that has never been operated on previously

Recurrent PD- A patient with PD who has had at least one previous operation

Recurrence – Recurrence of symptoms at a defined time point post operatively

Short tract – A pilonidal tract of <1cm. This can be a straight or curved tract or a midline or off-midline tract.

Simple PD- Patient with primary PD with a single tract

Success (rate) – Refers to achieving closure of sinus(es) and resolution of symptoms at least at 3 months post-operatively

Technique related Factors impacting failure or recurrence – Refers to failure to achieve complete hair control or sufficient brushing/diathermy of the tract intra-operatively
